# Modeling the transmission dynamics and control strategies during the 2017 diphtheria outbreak in Jakarta, Indonesia

**DOI:** 10.1016/j.idm.2025.08.004

**Published:** 2025-08-21

**Authors:** Bimandra A. Djaafara, Verry Adrian, Etrina Eriawati, Iqbal R.F. Elyazar, Raph L. Hamers, J. Kevin Baird, Guy E. Thwaites, Hannah E. Clapham

**Affiliations:** a Saw Swee Hock School of Public Health, National University of Singapore and National University Health System, Singapore, Singapore; b Oxford University Clinical Research Unit Indonesia, Faculty of Medicine Universitas Indonesia, Jakarta, Indonesia; c Jakarta Provincial Health Office, Jakarta, Indonesia; d Centre for Tropical Medicine and Global Health, Nuffield Department of Medicine, University of Oxford, Oxford, UK; e Oxford University Clinical Research Unit Vietnam, Ho Chi Minh City, Viet Nam

**Keywords:** Diphtheria, Outbreak, Susceptibility, Mathematical modelling

## Abstract

Diphtheria has resurged globally, including in Indonesia, despite widespread vaccination since the 1970s. Knowledge gaps persist in understanding contemporary transmission drivers and effective outbreak control, especially in densely populated areas like Jakarta. We analyzed the 2017 Jakarta outbreak data and developed a compartmental model incorporating estimates of population susceptibility and asymptomatic carriers. Key epidemiological parameters were estimated, and various control measures were simulated. Our study found overall diphtheria susceptibility at 12.9 % (95 % CrI: 8.6 %–19.0 %) and 28.0 % (95 % CrI: 20.5 %–36.0 %) in children under 5 under different modeling scenarios, which were below the 'herd immunity threshold'. We estimated asymptomatic carriers to be highly prevalent, substantially contributing to the reproduction number. The model indicated that contact tracing and treating suspected cases and their contacts were more effective in preventing new cases than catch-up vaccination alone. These findings provide valuable insights for future outbreak management strategies in similar settings.

## 1 Introduction

Before 1980, 1 million cases of diphtheria were estimated to occur each year worldwide, with 50,000–60,000 deaths (World Health Organization, 2017). The success of the Expanded Programme for Immunization (EPI) in the 1970s (Henderson, 1984) reduced the number of cases dramatically, and diphtheria seemingly disappeared from public awareness. Recently, diphtheria outbreaks have been reported in situations where humanitarian crises have disrupted the public health infrastructure (Dureab et al., 2019; Rahman & Islam, 2019). Additionally, nationwide outbreaks of diphtheria have also been reported in Venezuela (Zwizwai, 2017), and more recently in Nigeria (Adegboye et al., 2023). This global resurgence has extended to vulnerable groups in Europe, with an increasing number of diphtheria cases reported among asylum seekers and migrants (European Centre for Disease Prevention and Control, 2022; Hoefer et al., 2025). These varied contexts underscore that without sustained and comprehensive public health measures, diphtheria has the potential to reemerge as a major global threat. In Asia, the true burden of diphtheria, especially among children, is unknown due to weak or absent national surveillance systems (Nicholson et al., 2022).

Diphtheria is most commonly an air-borne infection caused by *Corynebacterium diphtheriae* that may lead to severe pharyngitis and potential airway obstruction. Asymptomatic or mild cases, serving as the main disease reservoir (Doull & Lara, 1925), are common. Diagnosis is primarily clinical but can be confirmed by the isolation of toxigenic *C. diphtheriae* from respiratory samples.

Vaccines, developed from diphtheria toxoid, confer 87 % protection against symptomatic disease after three or more doses (Truelove et al., 2020). Nevertheless, they do not protect against the asymptomatic carrier state (Grant, 1931), which may still be important transmitters in the community (Chen et al., 1985), particularly during periods of declining vaccination coverage. The extent of this role, however, remains unquantified. In Indonesia, diphtheria prevention primarily relies on the 3-dose pentavalent DPT-HB-Hib vaccine, administered to children from the age of 2 months at monthly intervals, followed by booster doses at various points of early childhood and during primary school (Direktorat Surveilans dan Karantina Kesehatan Kementerian Kesehatan Republik Indonesia, 2017).

In late 2017, Indonesia experienced a significant diphtheria outbreak that affected 28 provinces and 142 districts, including the capital city of Jakarta, which had long been spared from a major diphtheria outbreak. This event led to over 600 children being hospitalized and caused at least 38 recorded deaths (Dahuri, 2017). This alarming escalation represented a 42 % increase in cases at the national level compared to the previous year (Herriman, 2017). Low coverage rates for both primary and booster vaccinations were suspected as the primary driver of this national outbreak (Harapan et al., 2019).

In response to this crisis, Jakarta Provincial Health Office declared an outbreak of diphtheria at the beginning of December 2017, which prompted an Outbreak Response Immunization (ORI) campaign, targeting children up to 19 years old (Kementerian Kesehatan Republik Indonesia, 2017). During the first round of ORI (up to December 31, 2017), over 2.6 million children (92.1 % coverage) received their first booster vaccination dose. Contact tracing of suspected cases was also implemented and contacts were administered 14 days of erythromycin prophylaxis and checked for symptoms. Contacts with clinical symptoms or with lab confirmation also received diphtheria antitoxin (DAT) treatment.

While recent studies on diphtheria transmission modeling have made valuable contributions to understanding the disease's spread and control mechanism, significant knowledge gaps persist. In particular, there is a pressing need to understand the contemporary determinants of diphtheria transmission, especially in the context of declining vaccination coverage. We investigate the late 2017-early 2018 diphtheria outbreak in the densely populated metropolitan of Jakarta. Using modeling, we aim to estimate population susceptibility and key epidemiological parameters, understand the role of carriers on transmission, and estimate the effectiveness of control methods during the diphtheria outbreak in Jakarta.

## 2 Materials and methods

### 2.1 Epidemiological data

During the Jakarta diphtheria outbreak, the Jakarta Provincial Health Office collected data from individuals presenting symptoms aligned with the WHO's clinical description for suspected cases of diphtheria—an illness characterized by tonsillitis, laryngitis or pharyngitis, accompanied by a pseudomembrane on the tonsils, pharynx, and/or nose. These suspected diphtheria cases were primarily identified among individuals seeking treatment at primary health centers as well as contacts of these cases that were identified through contact tracing, which encompassed all household members of the affected individual and, if the case was a child, their neighboring friends. Of all the suspected cases, throat swabs were collected only from 60 (24 %), of which 18 (30 %) tested positive for *C. diphtheriae* culture. The analysis used the aggregated data of suspected diphtheria cases, separated by age groups to estimate population susceptibility, as well as the weekly aggregated data of suspected cases, based on their symptom onset dates, for the fitting of the mathematical model.

### 2.2 Estimating population susceptibility

We estimated the contact-adjusted initial population susceptibility (Funk et al., 2019) based on the distribution of suspected diphtheria cases by age groups during the four phases of outbreak in Jakarta (Phase 1: beginning of the outbreak; Phase 2: outbreak declaration; Phase 3: school holidays; and Phase 4: end of school holidays. Fig. S1 in **the Supplementary Information**). The population was divided into six age groups, which comprise four children age groups (0–4, 5–9, 10–14, and 15–19 years old) and two adult age groups (20–59, and 60+ years old). Those age groups were indexed as i=1,2,···,6. The four phases of the outbreak were indexed as k=1,2,3,4.

First, we calculate the force of infection ($\lambda_{i,k}$) experienced by each age group i during the phase k. The force of infection for age group i during phase k can be written as:

$$\lambda_{i,k}=\sum_{j=1}^{6}p_{\inf}\phi_{i,j}\frac{I_{j,k}}{N_{j,k}}$$

Where $p_{\inf}$ is the probability that a contact between a susceptible and infectious person resulting in an infection, $\phi_{i,j}$ is the number of contacts an individual of age group j makes with those from age group i per day (based on (Prem et al., 2021)), $I_{j,k}$ is the number of suspected diphtheria cases from age group j during phase k, and $N_{j,k}$ is the total population of age group j during phase k. Then, we calculated the number of cases in age group i during phase k ($c_{i,k}$) based on $\lambda_{i,k}$:

$$c_{i,k}=S_{i}\lambda_{i,k}N_{i}$$

Where $S_{i}$ is the initial population susceptibility of age group i. We then calculated the proportion of cases in age group i during phase k ($\pi_{i,k}$) as:

$$\pi_{i,k}=\frac{c_{i,k}}{\sum_{j=1}^{6}c_{j,k}}$$

The proportion of cases in each age group during phase k was fitted to the suspected diphtheria cases in each age group based on multinomial distribution:

$$I_{k}\;∼\;multinomial\left(\pi_{k}\right)$$

Two model assumptions for the prior distributions of the initial population susceptibility were used for the fitting process. Model 1 assumed an informed prior, Beta(30,90), for the 0–5 years old age groups based on the vaccination coverages from previous surveys (Badan Penelitian dan Pengembangan Kesehatan Kementerian Kesehatan RI, 2008, Badan Penelitian dan Pengembangan Kesehatan Kementerian Kesehatan RI, 2010, Badan Penelitian dan Pengembangan Kesehatan Kementerian Kesehatan RI, 2013, Badan Penelitian dan Pengembangan Kesehatan Kementerian Kesehatan RI, 2018) (Table 1) and assumed uninformed priors for the other age groups. Model 2 used the similar assumption for the youngest age group as Model 1, but also assumed informed priors of waning immunity due to lack of exposure and boosters, Beta(50,50), for the adult age groups. The model fitting process was done using the RStan (Stan Development Team, 2022) package in R software (R Core Team, 2022).

**Table 1:** The coverage of pentavalent DPT-HB-Hib basic vaccinations in children aged 12–23 months old in Jakarta.

Year	The coverage of three doses of DPT-HB-Hib	Source
2007	68.6	(Badan Penelitian dan Pengembangan Kesehatan Kementerian Kesehatan RI, 2008)
2010	62.5	(Badan Penelitian dan Pengembangan Kesehatan Kementerian Kesehatan RI, 2010)
2013	79.1	(Badan Penelitian dan Pengembangan Kesehatan Kementerian Kesehatan RI, 2013)
2018	72.9	(Badan Penelitian dan Pengembangan Kesehatan Kementerian Kesehatan RI, 2018)

### 2.3 Modeling framework

**Fig. 1:**
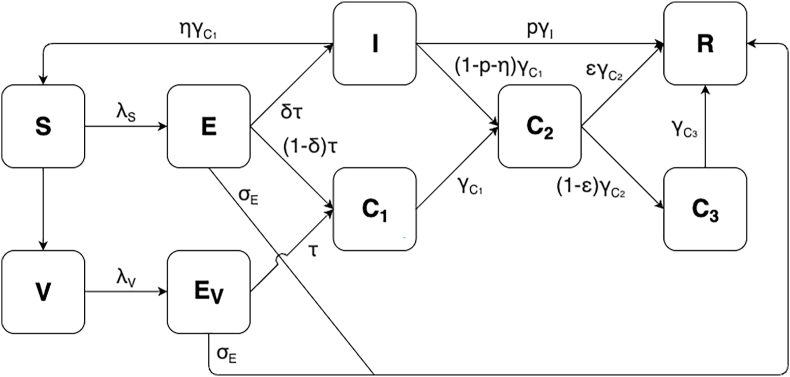
**Compartmental model diagram of diphtheria transmission dynamics.** The compartmental model accounts for the latent period of the disease (E), as well as the asymptomatic carriers (C_1_, C_2_, and C_3_) amongst the infected populations.

In our study, we developed a realistic compartmental model of diphtheria transmission, adapted from the model by Cvjetanovic et al. (Cvjetanović et al., 1978) (Fig. 1). The model was fitted to weekly aggregated data of suspected cases from the first week of November 2017 up to the first week of March 2018. This modeling framework accounts for the contributions of exposed individuals not yet symptomatic, symptomatic cases, and asymptomatic carriers. The latter was further categorized primary carriers (infected individuals who did not develop clinical symptoms), secondary carriers (untreated primary carriers), and chronic carriers (small fraction of secondary carriers that carry the bacteria longer).

Using this model, we estimated the key epidemiological parameters of the outbreak in Jakarta such as the basic reproduction number ($R_{0}$)—average number of secondary cases produced by an infectious individual in a fully susceptible population, and the effective reproduction number ($R_{t}$)—average number of secondary cases produced by an infectious individual in the population after accounting for population immunity and interventions implemented at time t. Furthermore, we evaluated the contribution of each infected population sub-group to the reproduction number value.

Our model included different phases that could potentially affect the transmission dynamics during the outbreak (Fig. S1). In each phase, we fitted model parameters to assess whether transmission was altered during each phase. Catch-up vaccination (ORI) and contact tracing were modeled by incorporating rate parameters that moved individuals from susceptible compartment to vaccinated compartment, and individuals from exposed compartment to recovered and immune compartment, respectively. Finally, the model fitting process was implemented using the RStan (Stan Development Team, 2022) package in R. Codes can be found at: https://github.com/andradjaafara/diphtheria_outbreak_jakarta.

### 2.4 Compartmental model structure

We used a diphtheria model structure based on Cvjetanovic et al. (Cvjetanović et al., 1978) with some modifications related to the interventions implemented during the outbreak in Jakarta. Upon infection, susceptible individuals (S) become infected and move to the incubation period (E). From E, individuals can develop into either infectious and symptomatic diphtheria cases or clinical diphtheria cases (I) or primary carriers ($C_{1}$) who are asymptomatic but still infectious. If caught by the surveillance system and given proper diphtheria treatment, infectious individuals move to fully recovered compartment (R) and therefore are immune to both diphtheria toxin and bacilli, or, if they did not get treatment, they become secondary carriers ($C_{2}$). From I, a small fraction of individuals who do not receive treatment become susceptible again (Dudley, 1922). Secondary carriers will be either fully recovered (R) or develop into chronic carriers ($C_{3}$). Those who were already vaccinated the beginning of the outbreak and those were vaccinated during the outbreak were denoted as V. Because vaccination is thought to only protect against disease, vaccinated individuals can still be infected and enter the incubation period $E_{V}$ with the same risk as the unvaccinated people (Frost et al., 1936). However, those vaccinated individuals would then only become asymptomatic primary carriers ($C_{1}$) (Miller et al., 1972). Immunity to diphtheria wanes over time (Grant, 1931) but we did not take this factor into account for the compartmental model as we only observed the outbreak progression for less than a year. Vaccination rates were adjusted so that from the start of the Outbreak Response Immunization (ORI) program until the end of February, all children were assumed to be vaccinated. The impact of contact tracing and treatment of contacts was incorporated to the model by adding an additional treatment recovery rate for exposed individuals ($\sigma_{E}$) and for primary carriers ($\sigma_{C}$). Fig. 1 shows the compartmental model diagrams used in this study.

The full compartmental model equations are as follows:

$$\frac{dS}{dt}=-\lambda S+\eta \gamma_{C_{1}}I-\rho S$$

$$\frac{dE}{dt}=\lambda S-\tau E-\sigma_{E}E$$

$$\frac{dI}{dt}=\delta \tau E-p\gamma_{I}I-\left(1-p\right)\gamma_{C_{1}}I$$

$$\frac{{dC}_{1}}{dt}=\left(1-\delta \right)\tau E+\tau E_{V}-\gamma_{C_{1}}C_{1}-\sigma_{C}C_{1}$$

$$\frac{{dC}_{2}}{dt}=\left(1-p-\eta \right)\gamma_{C_{1}}+\gamma_{C_{1}}C_{1}-\gamma_{C_{2}}C_{2}$$

$$\frac{{dC}_{2}}{dt}=\left(1-\varepsilon \right)\gamma_{C_{2}}C_{2}-\gamma_{C_{3}}C_{3}$$

$$\frac{dR}{dt}=p\gamma_{I}I+\varepsilon \gamma_{C_{2}}C_{2}+\sigma_{E}\left(E+E_{V}\right)+\sigma_{C}C_{1}$$

$$\frac{dV}{dt}=\rho S-\lambda V$$

$$\frac{{dE}_{V}}{dt}=\lambda V-\tau E_{V}-\sigma_{E}E_{V}$$

Where λ as the force of infection accounting for the contribution of symptomatic individuals, carriers, and individuals who are still in their incubation period but already shedding bacteria:

$$\lambda =\beta \times \kappa \times \frac{\left(I+\theta_{1}C_{1}+\theta_{2}\left(C_{2}+C_{3}\right)+\theta_{3}\left(E+E_{V}\right)\right)}{N}$$

### 2.5 Compartmental model parameters

The complete model parameters, denoting the rates and proportions for the movements between groups are presented in Table 2. Some parameters were fixed to certain values based on knowledge from previous studies, and others were estimated. Parameter values were carefully sourced from the best available evidence, including recent systematic reviews (Truelove et al., 2020), established epidemiological references for diphtheria (Anderson & May 1991; Cvjetanović et al., 1978), and contemporary diphtheria modeling study (Finger et al., 2019). Where multiple estimates were available, we selected values that were most appropriate for our study context. To accommodate the change in actions, policies, and behavior of the population during the outbreak, the outbreak was divided into four phases with parameter estimates during each phase were allowed to be different (Fig. S1).

**Table 2:** The list of compartmental model parameters and their prior distributions for model fitting.

Parameters	Symbol	Values	Value sources	Prior distributions
Basic reproduction number	$R_{0}$	Calculated	N/A	Gamma ∼ (37,1/14); based on Truelove et al. estimates (Truelove et al., 2020)
Transmission rate	β	Estimated	N/A	–
Susceptible proportion at the beginning of the outbreak	$S_{0}$	Estimated	N/A	Beta ∼ (21.66,143.12) for prior based on Model 1; Beta ∼ (63.28,106.12) for prior based on Model 2; Both were based on the initial susceptibility estimation results.
Progression rate from exposed to symptomatic infectious individuals or carriers	τ	1/3 days^−1^	(Anderson & May 1991; Finger et al., 2019)	–
Recovery rate of symptomatic infectious individuals during phase 2 and 3	$\gamma_{I}$	Phase 2 & 3: 1/(1.12 + 2) days^−1^; Phase 1, 4 & 5: 1/(3.88 + 2) days^−1^	Average difference between day of the onset of symptoms and admission to health centers plus two days of assumed full clearance of bacteria	–
Recovery rate of primary carriers, temporary carriers, chronic carriers, and symptomatic infectious individuals who did not get treated	$\gamma_{C_{1}},\gamma_{C_{2}},\gamma_{C_{3}}$	1/18 days^−1^	(Cvjetanović et al., 1978; Truelove et al., 2020)	–
Proportion of infected people who progress to become symptomatic infectious individuals	δ	0.70	Truelove et al. (2020)	–
Proportion of untreated symptomatic infectious individuals who become susceptible again	η	0.05	Cvjetanović et al. (1978)	–
Fraction of symptomatic cases reported	$p_{1}$ (Phase 1); $p_{2}$ (Phase 2–5)	Phase 1: Estimated; Phase 2, 3, 4 & 5: Estimated	N/A	$p_{1}$ - Beta ∼ (15,15)
				$p_{2}$ - Beta ∼ (27,6)
				$p_{2}$ was assumed to be higher than $p_{1}$
Reduction of infectiousness of acute carriers (C1)	$\theta_{1}$	Estimated	N/A	Beta ∼ (7.5,23); based on Truelove et al. estimates (Truelove et al., 2020)
Reduction of infectiousness of temporary and chronic carriers (C2 and C3)	$\theta_{2}$	Estimated	N/A	Beta ∼ (1,1)
Reduction of infectiousness of exposed individuals (E and E_V_)	$\theta_{3}$	Estimated	N/A	Beta ∼ (1,1)
Vaccination rate (assumed that around 100,000 children were vaccinated each day)	ρ	Assumed to be able to vaccinate all children in Jakarta by the end of February with a constant number of children vaccinated each day.	N/A	Beta ∼ (1,1)
Reduction of contact rate during high awareness period and school holidays	κ	Phase 2 & 3: Estimated; Phase 1, 4 & 5: 1	N/A	Beta ∼ (1,1)
Proportion of temporary carriers who become recovered and immune	ε	0.95	Truelove et al. (2020)	–
Treatment rate of exposed individuals as due to contact tracing	$\sigma_{E_{1}}$ (Phase 2–3); $\sigma_{E_{2}}$ (Phase 4–5)	Phase 1: 0; Phase 2 & 3: Estimated; Phase 4 & 5: Estimated	N/A	Beta ∼ (1,1) for both $\sigma_{E_{1}}$ and $\sigma_{E_{2}}$

### 2.6 Reproduction number calculation

The basic reproduction number ($R_{0}$), assuming a fully susceptible population, was divided into five components of contributors: 1) infectious and symptomatic (I); 2) exposed individuals (E); 3) primary carriers ($C_{1}$); 4) temporary carriers ($C_{2}$); and 5) chronic carriers ($C_{3}$). The formulas for each respective component of $R_{0}$ are shown below:

$$R_{0,E}=\frac{\theta_{3}\beta \kappa }{\tau }$$

$$R_{0,I}=\delta \left(\frac{\beta \kappa }{p\gamma_{I}+\left(1-p\right)\gamma_{C_{1}}}\right)$$

$$R_{0,C_{1}}=\left(1-\delta \right)\left(\frac{\theta_{1}\beta \kappa }{\gamma_{C_{1}}}\right)$$

$$R_{0,C_{2}}=\left(\delta \left(\frac{\left(1-p-\eta \right)\gamma_{C_{1}}}{p\gamma_{I}+\left(1-p\right)\gamma_{C_{1}}}\right)+\left(1-\delta \right)\right)\left(\frac{\theta_{2}\beta \kappa }{\gamma_{C_{2}}}\right)$$

$$R_{0,C_{3}}=\left(\delta \left(\frac{\left(1-p-\eta \right)\gamma_{C_{1}}}{p\gamma_{I}+\left(1-p\right)\gamma_{C_{1}}}\right)+\left(1-\delta \right)\right)\left(\frac{\theta_{2}\beta \kappa }{\gamma_{C_{3}}}\right)\left(1-\varepsilon \right)$$

$$R_{0}=R_{0,E}+R_{0,I}+R_{0,C_{1}}+R_{0,C_{2}}+R_{0,C_{3}}$$

The effective reproduction number ($R_{t}$) calculation accounts for the number of V and R individuals in the population, and adds the contribution of exposed vaccinated individuals into the transmission. The $R_{t}$ calculation is also modified by treatment of exposed individuals and carriers found during contact tracing, reduced within population contacts after the outbreak announcement and during the school holiday, and the proportion of the population with different immunity (S, V or R).

The $R_{t}$ calculation equations are as follows:

$$R_{t,E}=\left(\frac{S}{S+V+R}\right)\left(\frac{\theta_{3}\beta \kappa }{\tau +\sigma_{E}}\right)$$

$$R_{t,E_{V}}=\left(\frac{V}{S+V+R}\right)\left(\frac{\theta_{3}\beta \kappa }{\tau +\sigma_{E}}\right)$$

$$R_{t,I}=\delta \left(\frac{S}{S+V+R}\right)\left(\frac{\tau }{\tau +\sigma_{E}}\right)\left(\frac{\beta \kappa }{p\gamma_{I}+\left(1-p\right)\gamma_{C_{1}}}\right)$$

$$R_{t,C_{1}}=\left(\left(1-\delta \right)\left(\frac{S}{S+V+R}\right)\left(\frac{\tau }{\tau +\sigma_{E}}\right)+\left(\frac{V}{S+V+R}\right)\left(\frac{\tau }{\tau +\sigma_{E}}\right)\right)\left(\frac{\theta_{1}\beta \kappa }{\gamma_{C_{1}}+\sigma_{C}}\right)$$

$$R_{t,C_{2}}=\left(\delta \left(\frac{S}{S+V+R}\right)\left(\frac{\tau }{\tau +\sigma_{E}}\right)\left(\frac{\left(1-p-\eta \right)\gamma_{C_{1}}}{p\gamma_{I}+\left(1-p\right)\gamma_{C_{1}}}\right)+\left(\left(1-\delta \right)\left(\frac{S}{S+V+R}\right)\left(\frac{\tau }{\tau +\sigma_{E}}\right)+\left(\frac{V}{S+V+R}\right)\left(\frac{\tau }{\tau +\sigma_{E}}\right)\right)\left(\frac{\gamma_{C_{1}}}{\gamma_{C_{1}}+\sigma_{C}}\right)\right)\left(\frac{\theta_{2}\beta \kappa }{\gamma_{C_{2}}}\right)$$

$$R_{t,C_{3}}=\left(\delta \left(\frac{S}{S+V+R}\right)\left(\frac{\tau }{\tau +\sigma_{E}}\right)\left(\frac{\left(1-p-\eta \right)\gamma_{C_{1}}}{p\gamma_{I}+\left(1-p\right)\gamma_{C_{1}}}\right)+\left(\left(1-\delta \right)\left(\frac{S}{S+V+R}\right)\left(\frac{\tau }{\tau +\sigma_{E}}\right)+\left(\frac{V}{S+V+R}\right)\left(\frac{\tau }{\tau +\sigma_{E}}\right)\right)\left(\frac{\gamma_{C_{1}}}{\gamma_{C_{1}}+\sigma_{C}}\right)\right)\left(\frac{\theta_{2}\beta \kappa }{\gamma_{C_{3}}}\right)\left(1-\varepsilon \right)$$

$$R_{t}=R_{t,E}+R_{t,E_{V}}+R_{t,I}+R_{t,C_{1}}+R_{t,C_{2}}+R_{t,C_{3}}$$

### 2.7 Counterfactual scenario simulations

With the parameters estimated from the model fitting process (Model 1), we simulated a variety of counterfactual scenarios to assess the impact of incomplete and delayed control measures during the outbreak. These scenarios represent sub-optimal intervention strategies compared to the actual response. We performed simulations under the following conditions: 1) contact tracing as the only intervention during the outbreak (Scenario 1); 2) ORI as the only intervention during the outbreak (Scenario 2); and 3) ORI and contact tracing were implemented, but ORI was delayed by two weeks (Scenario 3). We calculated the differences in simulated diphtheria incidences between the actual interventions (implementation of both ORI and contact tracing) and the counterfactual scenarios, with positive values indicating additional cases that would have occurred under the alternative scenarios.

## 3 Results

### 3.1 Jakarta outbreak summary

**Fig. 2:**
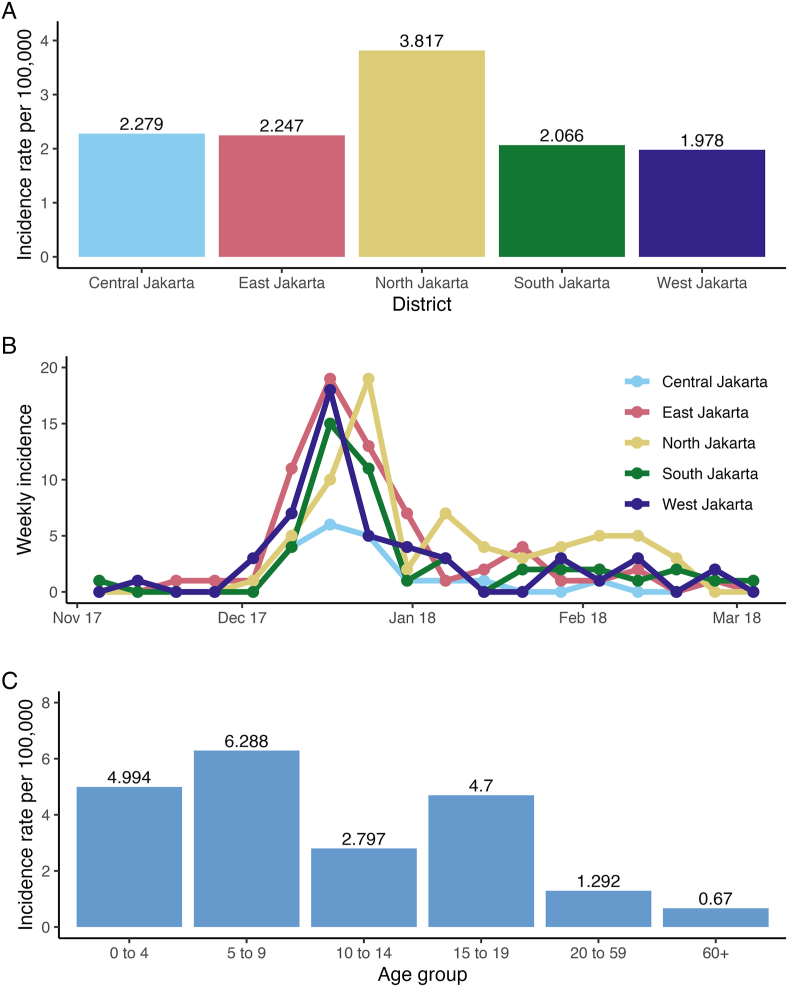
**Incidence rates of the diphtheria outbreak in Jakarta. A)** Incidence rate per 100,000 of suspected cases by district; **B)** Weekly reported suspected cases curves by district; **C)** Age-grouped incidence rate per 100,000 of suspected cases (244 out of 250 cases provided their age). The data cover the period from the first week of November 2017 to the first week of March 2018.

From November 2017 to March 2018, 250 suspected diphtheria cases were reported, peaking at 68 cases in the third week of December 2017. Two fatalities (0.8 % case fatality ratio/CFR) were recorded in November, preceding the vaccination campaign. North Jakarta had the highest incidence rate (3.82 per 100,000 people), nearly twice that of other districts (Fig. 2A). The epidemic curve shows incidence peaked in mid-December across all districts, with North Jakarta's peak trailing slightly. Following the government's outbreak declaration and the commencement of the school holiday in late December, incidence rates decreased dramatically (Fig. 2B).

The age distribution of the incidence rate during the outbreak is illustrated in Fig. 2C. Suspected cases predominantly comprised the 0–19 years old (62.8 %, 157/250 cases). The overall incidence rate in children was 4.76 per 100,000, almost quadruple the rate in adults (1.23 per 100,000). Among children, those aged 5–9 years had the highest incidence rate (6.29 per 100,000), followed by 15–19 years old (4.70 per 100,000).

Of the 97 cases (38.8 %) that could provide information on their primary vaccination status, 76 (78.4 %) reported complete primary diphtheria vaccinations. It should be noted, however, that not all individuals could present a vaccination record card as verification.

### 3.2 Susceptibility estimates

**Fig. 3:**
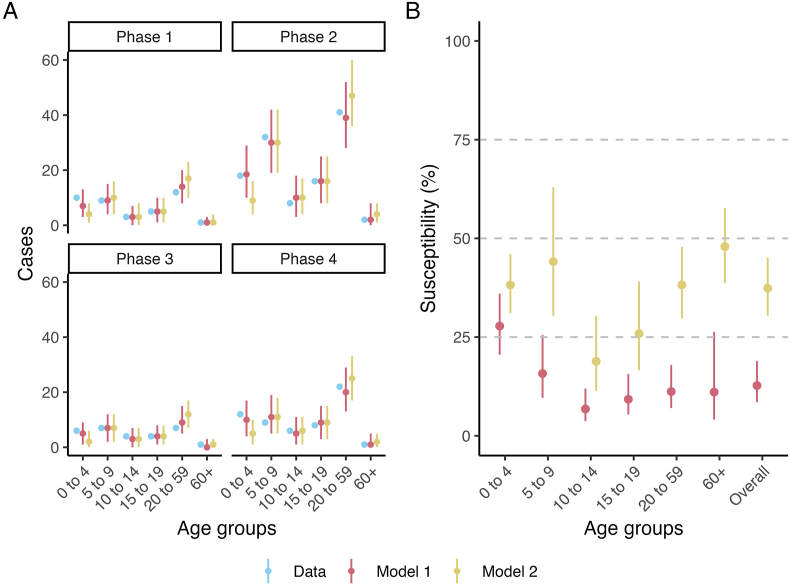
**Model fitting and estimates of susceptibility by age group. A)** Susceptibility model fitting to case data within the four phases of the outbreak for Model 1 and Model 2; and **B)** Initial population-level susceptibility to diphtheria estimates for all age groups and the overall population based on Model 1 and Model 2 based on the model fitting to case data (see **methods**).

We estimated the initial contact-adjusted population susceptibility to diphtheria at the start of the outbreak using two modeling assumptions (Fig. 3). Comparing the model fit to data between Model 1 and Model 2, we found that Model 1 has a better overall fit. In Model 2 fitting, we found that to compensate for higher susceptible assumptions in adults, the model estimated less cases in the youngest age groups (Fig. 3A). The age-grouped susceptibility profiles also differ between the two models (Fig. 3B). The overall susceptibility proportion based on Model 1 is 12.9 % (95 % CrI: 8.6 %–19.0 %). Based on Model 2, the overall population susceptibility is doubled: 28.0 % (95 % CrI: 20.5 %–36.0 %). The 0–4 years old age group has the highest susceptibility based on Model 1, while Model 2 estimated that 5–9 years old and the two adult age groups all have higher susceptibility proportions than 0–4 years old.

### 3.3 Epidemiological parameter estimates

**Fig. 4:**
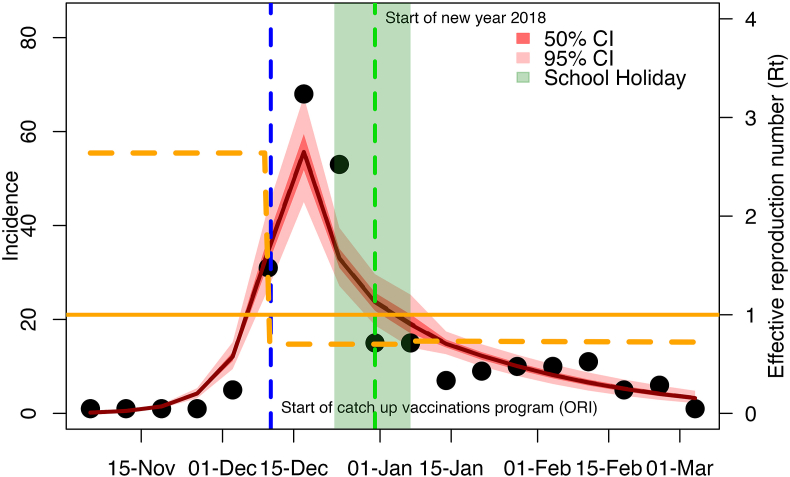
**Compartmental model simulations and fitting to weekly suspected case data.** Comparison of modeled weekly incidence (red line and the shaded areas as the credible intervals) based on the Model 1 estimates of initial population immunity to the observed weekly incidence (black dots). The orange dashed-line shows the estimated effective reproduction number over time ($R_{t}$). The solid orange line denotes the critical reproduction number threshold of 1.

From model fitting to data (Fig. 4 for the modeled trajectories based on Model 1 estimates of initial population immunity, and Fig. S2 of **the SI** for the one based on Model 2). The model managed to simulate the weekly suspected diphtheria cases trajectory during the outbreak period. The full description of all parameter estimates is available in Table 3 and Fig. 4 (trace plots of the MCMC fits are available at Fig. S3). Based on the parameter estimates, Model 1 and Model 2 estimates differed on the initial susceptibility estimates ($S_{0}$) and basic reproduction number ($R_{0}$). Estimates of other parameters are largely similar between the two model assumptions.

**Table 3:** Parameter estimates from each model fitting process. The scenario simulations on the main text uses the values based on Model 1.

Model	Parameters	Mean	Median	Lower CrI	Upper CrI
Model 1 (prior population immunity median of 12.94 %)	$S_{0}$	0.146	0.145	0.094	0.209
	$R_{0}$	3.961	3.946	3.471	4.526
	β	0.358	0.356	0.310	0.417
	$\theta_{1}$	0.198	0.196	0.085	0.324
	$\theta_{2}$	0.022	0.017	0.001	0.077
	$\theta_{3}$	0.928	0.944	0.760	0.998
	$p_{1}$	0.390	0.385	0.264	0.538
	$p_{2}$	0.863	0.869	0.748	0.946
	κ	0.339	0.325	0.220	0.540
	$\sigma_{E_{1}}$	0.043	0.028	0.001	0.168
	$\sigma_{E_{2}}$	0.796	0.819	0.464	0.991
Model 2 (prior population immunity median of 37.20 %)	$S_{0}$	0.393	0.392	0.322	0.466
	$R_{0}$	3.540	3.527	3.101	4.084
	β	0.305	0.301	0.263	0.369
	$\theta_{1}$	0.192	0.186	0.073	0.343
	$\theta_{2}$	0.034	0.026	0.001	0.115
	$\theta_{3}$	0.876	0.907	0.600	0.996
	$p_{1}$	0.322	0.316	0.215	0.454
	$p_{2}$	0.858	0.863	0.740	0.945
	κ	0.404	0.377	0.240	0.753
	$\sigma_{E_{1}}$	0.062	0.040	0.001	0.264
	$\sigma_{E_{2}}$	0.653	0.647	0.353	0.968

We estimated that exposed individuals had 94.4 % (95 % CrI: 76.0 %–99.8 %) of the infectiousness of symptomatic individuals ($\theta_{3}$), primary carriers exhibited 19.6 % (95 % CrI: 8.5 %–32.4 %) infectiousness ($\theta_{1}$), and temporary and chronic carriers showed only 1.7 % (95 % CrI: 0.1 %–7.7 %) infectiousness ($\theta_{2}$). Initially, we estimated that surveillance was only able to capture 38.5 % (95 % CrI: 26.4 %–53.8 %) of clinical cases, which then improved to 86.9 % (95 % CrI: 74.8 %–94.6 %).

The median estimated basic reproduction number ($R_{0}$) for the outbreak, based on the Model 1, was 4.0 (95 % CrI: 3.5–4.5), which fell within the value range of the prior distribution used for the model fitting. The initial susceptible population proportion median estimate is 14.5 %, slightly higher than the prior median of ∼13.0 %, but the prior is still well within the estimated 95 % CrI of 9.4 %–20.9 %. Early in the outbreak, the estimated effective reproduction number ($R_{t}$) value, which accounts for population immunity, was 2.6 (95 % CrI: 2.1–3.0), around 30 % lower than the $R_{0}$. The major contributor to the $R_{t}$ value was asymptomatic carriers at 50.5 %, whereas symptomatic cases and exposed individuals contributed only 8.5 % and 41.0 %, respectively to the overall $R_{t}$ (Fig. 6A). Our calculations show that the contribution of asymptomatic carriers and symptomatic cases to the $R_{t}$ of diphtheria is related to the level of population immunity (Fig. 6A). In addition, we estimated a decline in the $R_{t}$ value as the level of population immunity increased (Fig. 6B), however, the $R_{t}$ would not reach values lower than 1, even if the whole population was immune. The proportion of clinical cases decreases when the level of population immunity is high (Fig. 6C), implying a reduction of clinical burden.

**Fig. 5:**
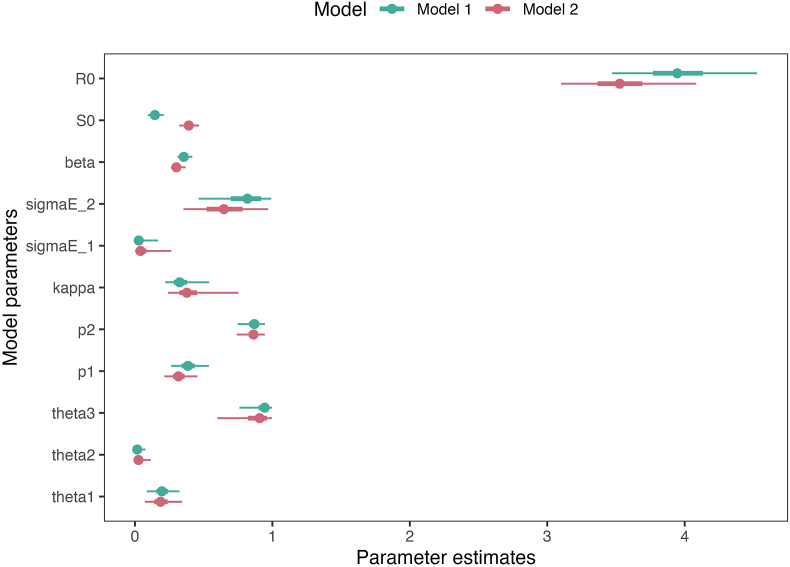
Median, 50 % Credible Intervals, and 95 % Credible Intervals of all the estimated model parameters based on the MCMC. The estimates shown are based on both Model 1 and Model 2 priors of initial population immunity.

**Fig. 6:**
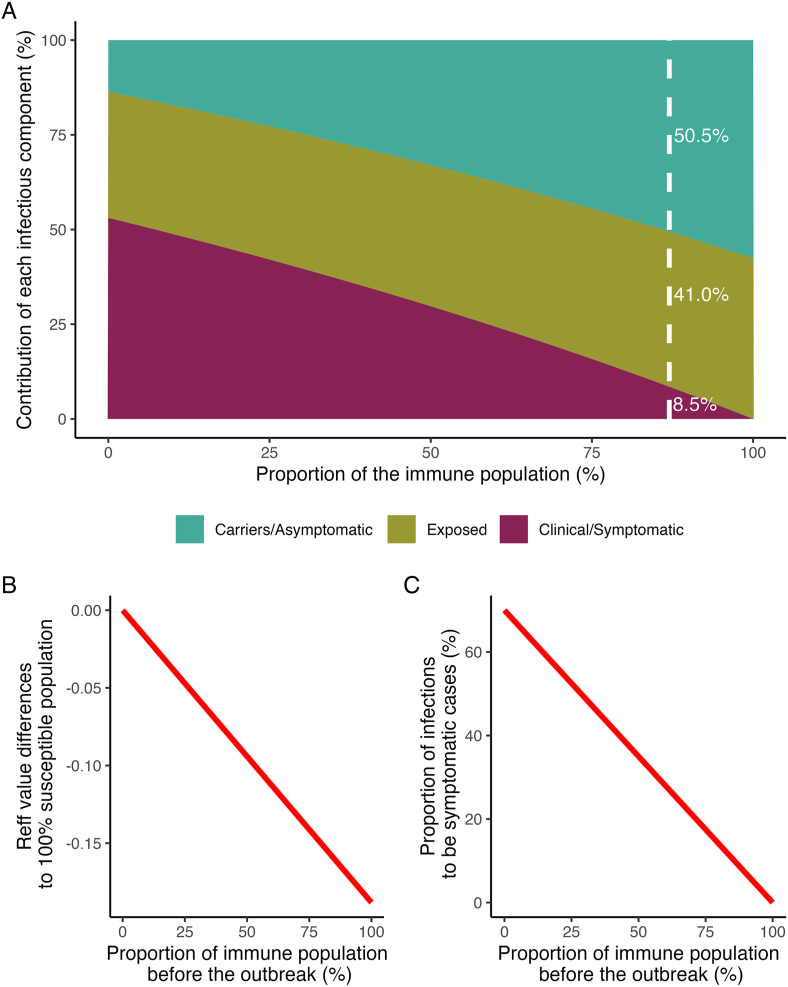
**Relative contributions of each disease state towards diphtheria reproduction number and the estimated effects of the proportion of the immune population on reproduction number and proportion of symptomatic cases. A)** The relationship between the population immunity to diphtheria and the contribution of each infectious component to the reproduction number of diphtheria. The white dashed line denotes the estimated population immunity level at the beginning of the 2017 diphtheria outbreak in Jakarta, based on Model 1; **B)** The relationship between the initial population immunity and the differences between the outbreak reproduction number compared to the basic reproduction number; and **C)** The relationship between the initial population immunity and the proportion of clinical cases emerging during an outbreak.

We observed a dramatic decline in $R_{t}$ following the outbreak declaration in December and during the school holiday, largely attributed to the estimated 32.5 % (95 % CrI: 22.0 %–54.0 %) decrease in the transmission rate (κ). The $R_{t}$ value increased to 0.7 after the school holiday was over. However, this remained below the crucial threshold of 1, which resulted in slow continued decline in case numbers.

### 3.4 Counterfactual scenario simulations

**Fig. 7:**
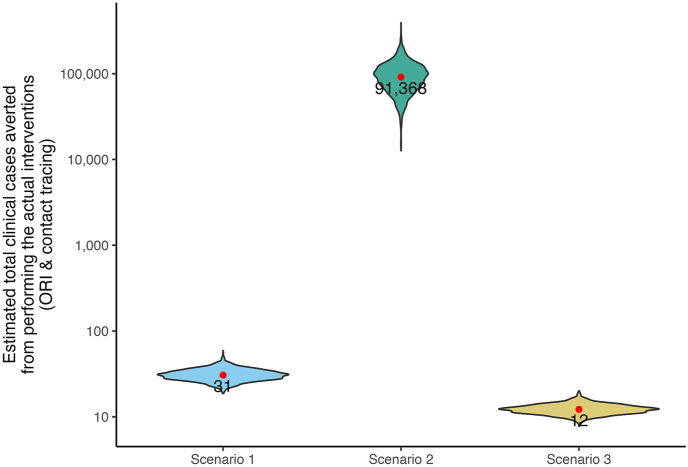
**Estimated total diphtheria clinical cases averted based on different simulation scenarios.** Scenario 1: Contact tracing as the only intervention during the outbreak. Scenario 2: ORI as the only intervention during the outbreak (Scenario 2); Scenario 3: ORI and contact tracing were implemented, but ORI was delayed by two weeks.

We simulated three counterfactual scenarios that represented incomplete or delayed control measures during the outbreak to assess the impact of catch-up vaccination (ORI) and contact tracing (Fig. 7). Our simulation results suggest that if only contact tracing had been implemented without ORI (Scenario 1), there would have been 31 additional diphtheria cases (CrI 95 %: 23–42). Our estimates also suggest that if ORI had been conducted without contact tracing (Scenario 2), there could have been 90,000 additional cases (CrI 95 %: 37,149–184,103). Furthermore, we estimated that delaying ORI by two weeks while maintaining contact tracing (Scenario 3) would have resulted in only 12 additional cases (CrI 95 %: 10–16).

## 4 Discussion

In 2017, Indonesia experienced a large diphtheria outbreak, with the onset of suspected cases in Jakarta increasing in November and reaching a peak in mid-December, partly due to the control measures implemented by the Jakarta Health Office. Diphtheria was reported in every district in Jakarta, with North Jakarta reporting rates nearly twice that of other districts. The outbreak predominantly affected children in the 5-9- and 15-19-year age groups. The overall population immunity level of 87 %, equivalent to 13 % overall susceptibility, was close to the accepted herd immunity threshold. However, in children under 5 years, the proportion of children with immunity was estimated to be very low at 72 % (28 % susceptibility proportion). Control measures, such as contact tracing and treating suspected cases and their contacts, were pivotal in managing the outbreak and were considerably more effective in averting additional cases than catch-up vaccination (ORI) alone. Lastly, our estimations of the reproduction numbers ($R_{0}$ and $R_{t}$) largely aligned with recent pooled analysis (Truelove et al., 2020), with median $R_{0}$ of 4.0 (falls within 1.7–4.3 range (Truelove et al., 2020)) and median initial growth phase $R_{t}$ of 2.6 (falls within 1.1–3.2 range (Truelove et al., 2020)). However, we discovered that although asymptomatic carriers were individually less susceptible, they contributed substantially to the overall reproduction number, highlighting the substantial role asymptomatic carriers played during the outbreak.

Our estimated Jakarta immunity profile, where adults have higher protection compared to school-aged children, is similar to data from Nha Trang City in Vietnam, where primary vaccination coverage has been very high and no diphtheria cases have been reported in the city since 2013 (Kitamura et al., 2023). The difference with Jakarta is that the protection in early childhood was much lower in Jakarta than in Nha Trang. By contrast, the relatively high level of protection in 10–14 years old in Jakarta may reflect school-age booster campaigns (around 50 % coverage (Badan Penelitian dan Pengembangan Kesehatan Kementerian Kesehatan RI, 2018), which were not provided in Nha Trang.

Before the 2017 outbreak, Jakarta had not experienced any major diphtheria outbreaks in the recent decade. The vaccination coverage for the basic three-dose DPT in Jakarta had improved, rising from 68.6 % in 2007 to 79.1 % in 2013, but subsequently declined to 72.9 % by 2018 (Table 1). These coverages remained below the 90 % coverage target and the critical 80–85 % threshold necessary for ‘herd immunity’. No comprehensive research explains the low vaccination coverage specifically in Jakarta, but national-level studies indicate that low vaccination coverage may be attributed to socioeconomic factors (Herliana & Douiri, 2017). The prolonged period during which vaccination coverage remained below the critical threshold may have set the stage for a significant diphtheria outbreak in Jakarta.

We also estimated that diphtheria susceptibility was low amongst adults despite it is well known that protection against diphtheria wanes over time if boosters are not given (Swart et al., 2016). Sunarno et al. found that isolates of diphtheria cases in Jakarta to be closely related to those from the surrounding provinces, highlighting the role of population mobility in diphtheria spreads (Sunarno et al., 2020). Considering the significantly lower primary vaccination coverage in surrounding provinces like Banten and West Java, it is plausible that diphtheria had been quietly circulating among the highly-mobile adult population, thereby preserving their immunity. This silent circulation may explain the observed high protection among adults. Additionally, the circulation of undetected and unreported cutaneous diphtheria, including with non-toxigenic strains commonly circulating in highly-immunized populations (Gunatillake & Taylor, 1968), may be another possible reason for a maintained high population immunity to diphtheria in Jakarta.

Interestingly, our analysis showed that contact tracing was more effective in preventing cases compared to the catch-up vaccination campaign in children (ORI). We acknowledge that our model's homogeneous mixing assumption may contribute to overestimating the magnitude of projected case increases in some scenarios, particularly when interventions are implemented in isolation. Nonetheless, contact tracing has the added benefit of enabling the treatment and isolation of potentially exposed individuals. This aspect is crucial in suppressing the further spread of diphtheria by asymptomatic carriers within the population, highlighting the dual importance of contact tracing and vaccination in controlling diphtheria outbreaks, especially within the context of an urban outbreak with a relatively high population immunity. Our findings are similar to the pooled analysis by Truelove et al. (Truelove et al., 2020), highlighting the heightened importance of contact tracing compared to ORI during outbreak situations. Nonetheless, the catch-up vaccination campaign (ORI) targeting children, who are the most vulnerable group, remains a critical strategy during such outbreaks.

Finally, we found that the case fatality ratio (CFR) during the Jakarta outbreak was very low (0.8 %) compared to the overall CFR across the country in 2017 (4.7 %) (Karyanti et al., 2019), and the finding from a hospital-based study in Jakarta and Tangerang (3.5 %) (Arguni et al., 2021). This may be due to the broad inclusion of suspected diphtheria cases, which may include those within the lower spectrum of disease severity.

Our study has several limitations. First, we used suspected diphtheria data reported by the surveillance system, not confirmed cases, due to the challenging task of linking the laboratory results and surveillance data (Pane et al., 2023). Despite this, the specificity of diphtheria symptoms allows us to maintain confidence in our modeling outcomes. Secondly, there is a lack of information on the prior immunity level in adults; hence, the population susceptibility estimates were generally based on the number of cases reported in those age groups and the degree of connectedness based on a contact matrix. Some model parameters were also sourced from older studies, highlighting the need for contemporary estimates to reflect current conditions. Lastly, our analysis overlooked geographical variations in susceptibility that could influence provincial transmission dynamics. As an example, despite a reported >90 % ORI coverage across the province, a study in a sub-district in North Jakarta found that the ORI coverage was just around 60 % (Arfimita & Surjono, 2020). Finally, our compartmental model assumed homogeneous mixing, which may simplify the transmission process. This assumption may particularly affect our counterfactual scenario estimates, such as the substantial case increase projected for Scenario 2 (ORI without contact tracing), where the magnitude of effect may be overestimated due to the model's inability to capture spatial and social heterogeneity in real-world vaccination coverage and transmission patterns. However, due to the small amount of information and data for the model fitting process coupled with the complexity of the model, a more complex model incorporating age- and socioeconomic-mixing may overfit the data. For sensitivity analysis, we also tested the model simulations using the higher estimates of susceptibility based on Model 2, which gives us similar estimates in key epidemiological parameters (Fig. 5 and Table 3), increasing the confidence on our parameter estimates.

Despite the limitations, our results largely align with recent pooled analyses of diphtheria's epidemiological parameters, as conducted by Truelove et al. (Truelove et al., 2020) Our findings highlight that Jakarta's population immunity, especially among children, was below the ‘herd immunity’ threshold. Outbreak responses, particularly contact tracing of suspected cases, helped curb a larger outbreak in the community. Future research should focus on comprehensive seroprevalence and diphtheria carriage surveys in Jakarta to understand the disease's behavior in a highly-mobile urban population outside of an outbreak setting, and potential cross-circulation of diphtheria between Jakarta and the surrounding provinces. Investigating the social and behavioral factors contributing to the low vaccination coverage and population immunity in children to identify barriers and enable more targeted health interventions will also be crucial future studies.

## CRediT authorship contribution statement

**Bimandra A. Djaafara:** Writing – review & editing, Writing – original draft, Methodology, Formal analysis, Conceptualization. **Verry Adrian:** Writing – review & editing, Data curation, Conceptualization. **Etrina Eriawati:** Writing – review & editing, Data curation, Conceptualization. **Iqbal R.F. Elyazar:** Writing – review & editing, Conceptualization. **Raph L. Hamers:** Writing – review & editing, Conceptualization. **J. Kevin Baird:** Writing – review & editing, Conceptualization. **Guy E. Thwaites:** Writing – review & editing, Conceptualization. **Hannah E. Clapham:** Writing – review & editing, Writing – original draft, Methodology, Conceptualization.

## Disclaimer

The views expressed are those of the authors and should not be construed to represent the position of any of the institutions mentioned above.

## Ethical approval statement

No human subjects were involved in this study, so ethical approvals were not required for this manuscript.

## Funding source

This work was supported by Wellcome Trust. HEC is supported by NUS Start up Grant.

## Declaration of competing interest

The authors declare that they have no known competing financial interests or personal relationships that could have appeared to influence the work reported in this paper.

The author is an Editorial Board Member/Editor-in-Chief/Associate Editor/Guest Editor for *Infectious Disease Modelling* and was not involved in the editorial review or the decision to publish this article.
