# Supplementary material for: Modeling the transmission dynamics and control strategies during the 2017 diphtheria outbreak in Jakarta, Indonesia
Source: Infect Dis Model. 2025 Aug 21;11(1):1–15. doi: 10.1016/j.idm.2025.08.004 (PMC12454891; doi:10.1016/j.idm.2025.08.004)
Supplement: Multimedia component 1 [file mmc1.docx]

# **Supplementary Information**


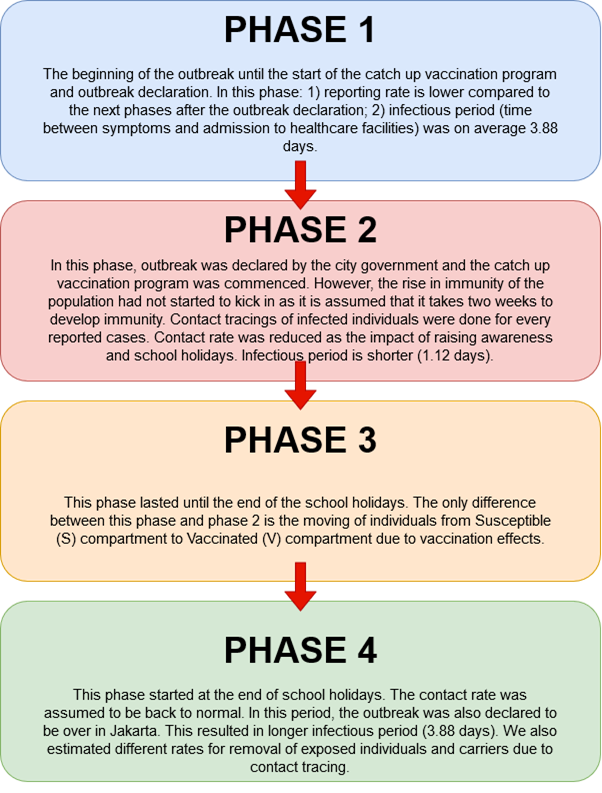


**Figure S1.** Diphtheria outbreak phases that affected modeling assumptions for model fitting and simulations.


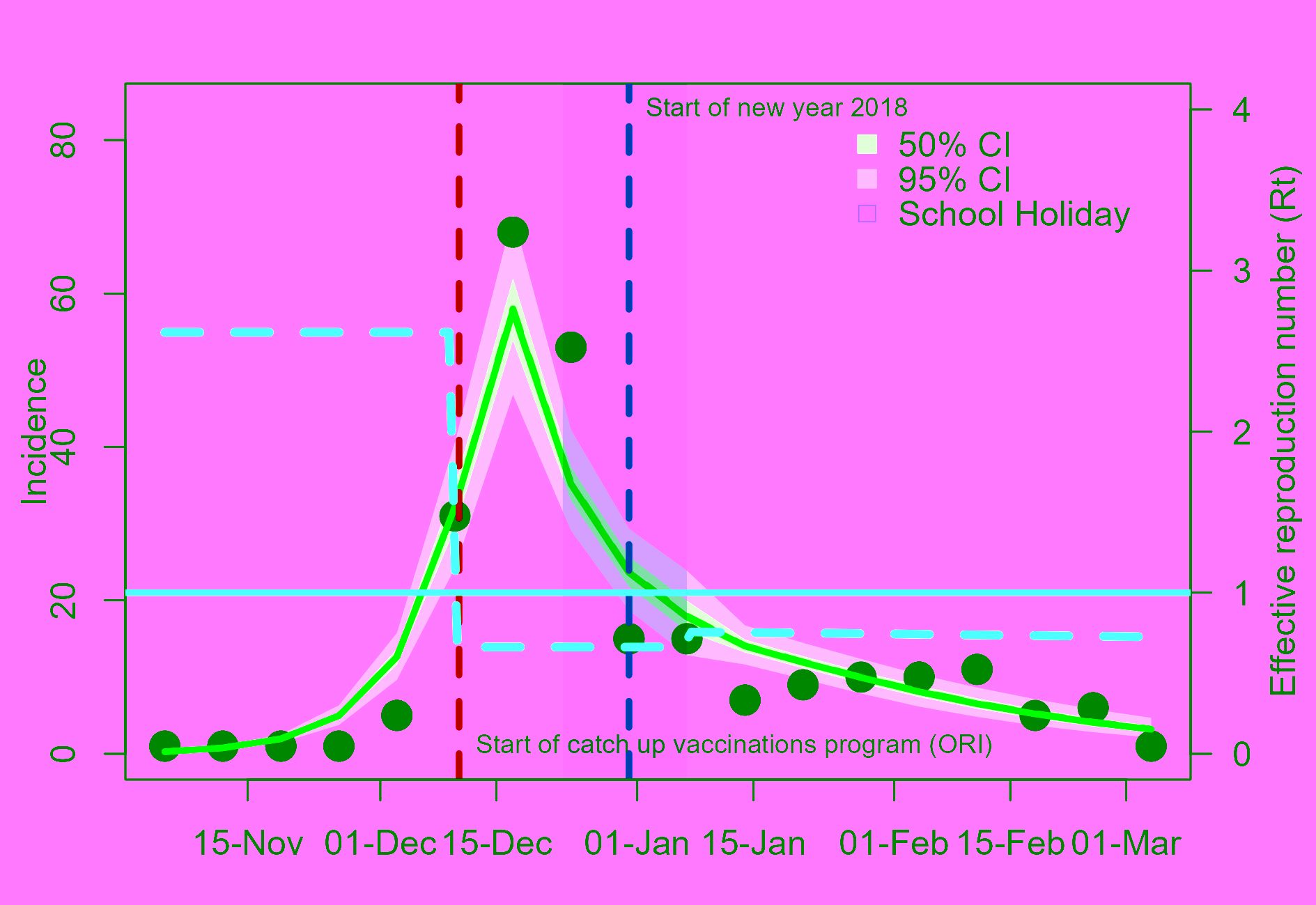


**Figure S2.** Comparison of modeled weekly incidence (red line and the shaded areas as the credible intervals) based on the Model 2 estimates of the initial population immunity to the observed weekly incidence (black dots). The orange dashed line shows the estimated effective reproduction number over time ($R_{t}$). The solid orange line denotes the critical reproduction number threshold of 1.


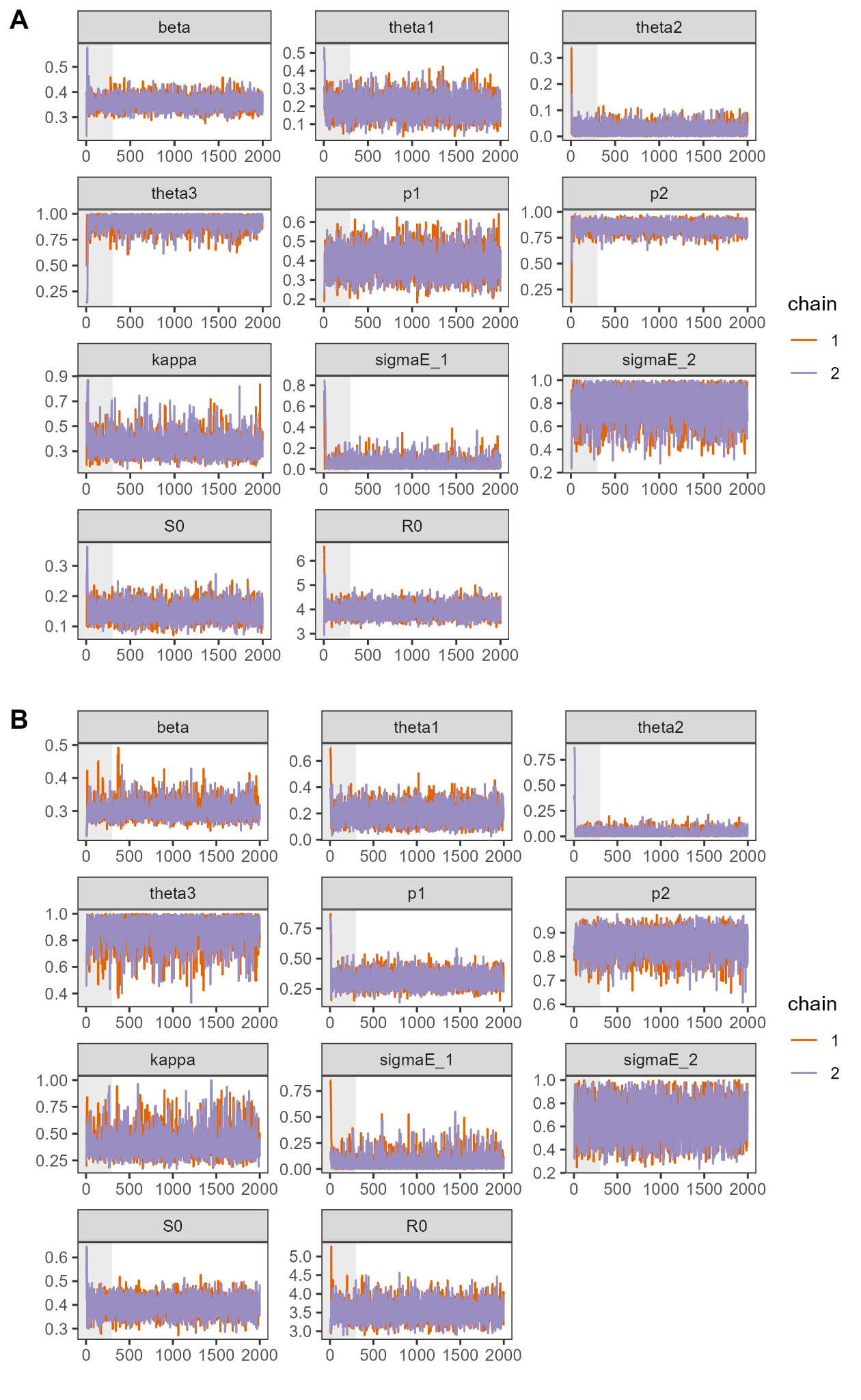


**Figure S3.** Trace plots of the MCMC run for: **A)** model based on Model 1 prior of the initial population immunity; and **B)** model based on Model 2 prior of the initial population immunity.
